# A rapid review of the effects of GLP-1 receptor agonists on opioid and stimulant use-related outcomes

**DOI:** 10.1016/j.dadr.2026.100440

**Published:** 2026-04-24

**Authors:** Marisa Leach, Megan E. Marziali, Surita Parashar, Greg Bondy, Silvia Guillemi, Marianne Harris, Mark Hull, Sarah Stone, Katherine W. Kooij, Robert S. Hogg, Julio S.G. Montaner

**Affiliations:** a Faculty of Health Sciences, Simon Fraser University, Burnaby, British Columbia, Canada; b British Columbia Centre for Excellence in HIV/AIDS, St. Paul’s Hospital, Vancouver, British Columbia, Canada; c Department of Epidemiology, Columbia University Mailman School of Public Health, New York, NY, United States; d Faculty of Medicine, University of British Columbia, Vancouver, British Columbia, Canada; e John Ruedy Clinic, St. Paul’s Hospital, Vancouver, British Columbia, Canada; f Diabetes Clinic, St. Paul’s Hospital, Vancouver, British Columbia, Canada; g Infectious Diseases Clinic, St. Paul’s Hospital, Vancouver, British Columbia, Canada

**Keywords:** Stimulant use disorder, Opioid use disorder, Glucagon-like peptide-1 receptor agonist

## Abstract

**Aim:**

Drug poisonings involving opioids and/or stimulants are a major public health concern. Pharmaceutical interventions to treat substance use disorders, particularly those involving stimulants, are limited. Glucagon-like peptide-1 receptor agonists (GLP-1RAs), approved to manage conditions such as type 2 diabetes, have been observed to reduce substance use as a secondary effect. We examined the current literature on GLP-1RAs as a potential treatment for either stimulant or opioid use disorder.

**Methods:**

We conducted an electronic search in PubMed, PsycINFO, Scopus, and Web of Science in September 2025 and January 2026 to identify studies investigating GLP-1RA treatment for stimulant or opioid use disorder. We identified 630 unique search results. One reviewer screened the title, abstract, and the full-text of results before performing data extraction. Metrics synthesized included study design, population characteristics, dosage information, and substance use behaviours or outcomes.

**Results:**

We identified eighteen studies between animal (n = 14) or human (n = 4) participants and stimulant- (n = 6) or opioid-related (n = 12) outcomes. Research conducted in the United States represented the majority of studies (n = 13). Overall, sixteen studies found a relationship between GLP-1RA administration and reduced stimulant- or opioid-related outcomes, three observing greater reduction in groups with higher GLP-1RA dosages or more extreme substance use at baseline. One study identified the incidence rate ratio of opioid overdose as 40% lower for people with a GLP-1 RA prescription compared to those without.

**Conclusion:**

These preliminary findings support that GLP-1RAs may be a potential pharmaceutical treatment for opioid or stimulant use disorders. Further research in human populations is needed.

## 1 Introduction

Drug poisoning deaths (i.e., overdoses) constitute a public health emergency in the United States and Canada, with incidence sharply escalating in the past ten years (Health Canada, Public Health Agency of Canada & U.S. Department of Health and Human Services, 2022). In Canada, the rates of opioid and stimulant toxicity have nearly doubled since 2019, reaching 17.7 and 17.1 deaths per 100,000 population in 2024, respectively (Substance-related Overdose and Mortality Surveillance Task Group on behalf of the Council of Chief Medical Officers of Health, 2025). Nonfatal overdoses contribute significantly to the overdose crisis. Per month, the US Centers for Disease Control and Prevention estimates 12.4 opioid-related and 2.6 stimulant-related nonfatal overdoses per 10,000 emergency department visits in the United States (Centers for Disease Control and Prevention, 2026). In British Columbia (BC), Canada, emergency dispatchers received 621,373 emergency calls in 2025 for all instances of overdose, a 15% increase over the past five years (Provincial Health Services Authority, n.d.). There is a pressing need in the US and Canada to address rates of fatal and nonfatal overdoses attributable to opioids and stimulants.

Few pharmaceutical interventions are available to effectively treat substance use disorders, particularly stimulant use disorder; there are currently no approved treatments for stimulant use disorder (Dowell et al., 2024; Opioid Use Disorder and Treatment, 2023; Stimulant Use Disorder Practice Update, 2022). Although safe and effective treatment for opioid use disorder is available, including methadone and buprenorphine, uptake is suboptimal: only one quarter of adults in the United States with opioid use disorder received medication in 2022, and a large study in BC reported only 33% of those with opioid use disorder were receiving pharmacological treatment (Dowell et al., 2024; Opioid Use Disorder and Treatment, 2023; Piske et al., 2020). Nonpharmaceutical treatments for substance use disorders (e.g., counseling, behavioural therapies) exist but require more time, effort, and support from trained personnel, with individual costs exceeding those of pharmaceutical treatments (French et al., 2008, Substance Abuse and Mental Health Services Administration, 2025). Even with these other forms of treatment, unmet need for treatment is high, leaving 1.8 million adults with a substance use disorder in the United States wanting but not receiving treatment in 2024 (Substance Abuse and Mental Health Services Administration, 2025). Underscoring this lack of support, the proportion of people returning to drug use after treatment reaches upwards of 60% among people with either an opioid or stimulant use disorder (Brecht and Herbeck, 2014, Nunes et al., 2018). Novel, effective interventions are needed for people with stimulant and/or opioid use disorders.

Glucagon-like peptide-1 receptor agonists (GLP-1RAs) potentially have promise as novel pharmaceutical therapies for substance use disorders. GLP-1RAs stimulate functions of GLP-1, which is a protein that binds to GLP-1 receptors to manage glucoregulatory processes (Liu et al., 2025). GLP1-RAs, through binding to the GLP-1 receptors, signal increased insulin production, decreased glucagon release, and control stress and reward processing, among other functions (Liu et al., 2025). On a larger scale, this signaling reduces appetite and food intake (Liu et al., 2025). Research with GLP-1RAs, currently approved to manage type 2 diabetes, weight loss, cardiovascular disease and chronic kidney disease in countries such as the United States and Canada, suggests that a secondary effect of GLP-1RA use is reduced substance use, potentially through feelings of satiation (Brunchmann et al., 2019, Canada’s Drug Agency-L’Agence de médicaments du Canada, 2022, Office of the Commissioner, 2024). Additional research has shown that GLP-1RAs interact with reward mechanisms and cognition in the brain, another pathway that impacts cravings and motivation to use alcohol or other substances (Arillotta et al., 2024, Chuong et al., 2023). Though these changes have been observed across the GLP-1RA drug class as a whole, differing half-lives, routes of administration, and bond strength to GLP-1 receptors influence the ease of use and efficacy for each analog (Schuckit, 2009).

In this study, we aimed to explore the literature and synthesize available information on GLP-1RAs as a potential treatment for opioid and stimulant use, considering both animal- and human-based studies. By exploring evidence presented in these studies, we aim to identify gaps in research to inform future research priorities. Our ultimate goal is to strengthen the evidence base concerning GLP-1RAs as a potential intervention for opioid use disorder and stimulant use disorder.

## 2 Methods

We conducted this rapid review adhering to approaches suggested by King et al. (2022) and following the PRISMA Scoping Review Guidelines where applicable (Tricco et al., 2018). Each author reviewed and revised the protocol for this review. The final protocol was published via the Open Science Framework (Leach et al., 2025).

### 2.1 Search strategy

We developed a PICO-based search strategy (Population, Intervention, Comparison to intervention, and Outcomes of interest) using relevant search terms and controlled vocabulary, seen in Table 1 (What Is PICO?, n.d.). An example search is provided in Table S1. All authors reviewed and edited search terms where applicable. Exact searches varied according to each database. We conducted the initial search in PubMed, PsycINFO, Scopus, and Web of Science in September 2025; we updated the search in January 2026, following the same procedure to ensure search results remained relevant.

**Table 1:** Search strategy and search terms applied across databases.

**Search term category**	**Search terms**	**Type of term**
GLP-1s	Agonists, glp 1r; agonists, glp1r	MeSH terms
	semaglutide; lixisenatide; albiglutide; dulaglutide; liraglutide; exenatide; ozempic; wegovy; trulicity; victoza; saxenda; mounjaro; adlyxin; eperzan; byetta; bydureon; zepbound; tanzeum; rybelsus; soliqual; xultrophy	Title/abstract
Substance use	opioid*; opiate*; OUD; fentanyl; oxycodone; heroin; morphine; cocaine; CUD; stimulant*; methamphetamine; amphetamine	Title/abstract

Study eligibility included: (1) written in English, (2) operationalized the exposure as a GLP-1RA, which could include the drug class as a whole or differentiation between analogs (i.e., semaglutide, lixisenatide, albiglutide, dulaglutide, liraglutide, tirzepatide, or exenatide), and (3) operationalized the outcome as a stimulant or opioid use-related outcome. Stimulant or opioid use-related outcomes could include metrics about changes to drug use and craving, or measures of health outcomes such as overdoses and hospitalizations. We included (4) studies where the population included animal models or human participants, and (5) original, empirical studies. Only published articles met our inclusion criteria. We excluded studies if they were exclusively qualitative, case reports, or reviews, as well as grey literature. Our review also excluded studies that did not isolate stimulant- or opioid-related outcomes. Included studies could be published in any year.

**Fig. 1:**
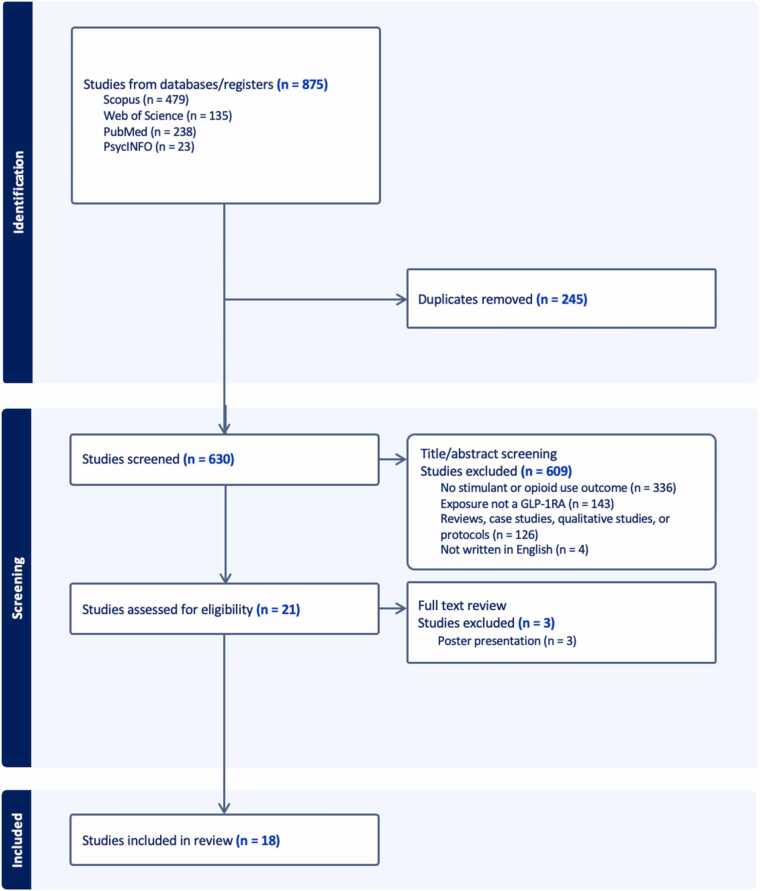
Screening flowchart.

We uploaded all search results to Covidence, which automatically removed duplicates as shown in Fig. 1 (Veritas Health Innovation, n.d.). In total, our search yielded 875 results and 245 duplicates. With the resulting 630 manuscripts, one reviewer (ML) followed eligibility criteria to screen titles and abstracts. Of this pool, one reviewer (ML) marked 609 results irrelevant, the primary reasons being that works did not include stimulant or opioid use-related outcomes (n = 336), or that results did not operationalize GLP-1RAs as an exposure (n = 143). The reviewer then completed a full-text review of 21 studies to thoroughly examine for eligibility. Full reasons for exclusion are presented in Fig. 1. We opted to have one reviewer screen for eligibility and extract data in accordance with methodologies applied in previous rapid reviews (Alur et al., 2024; King et al., 2025; King et al., 2022; Potvin et al., 2024; Tricco et al., 2015).

### 2.2 Data extraction

One reviewer (ML) extracted data from all included studies according to Cochrane’s data collection process for systematic reviews to establish a standardized procedure (Li et al., 2019, Chapter 5). This guideline informed the metrics collected and tools used to ensure that the same data were recorded for each study. Two authors (ML, MEM) developed a data extraction template for both human and animal studies prior to extraction (Table S2). One reviewer (ML) independently carried out extraction using this template formatted in Covidence. Extracted data included publication details (e.g., author, date of publication, and country of origin), sample size and population characteristics (e.g., sex, inclusion and exclusion criteria), methods (e.g., study design, dosage information, substance use measure, and statistical methods of analysis), and the study objective and findings.

### 2.3 Synthesis

We compiled data for studies investigating stimulant use and opioid use. We distinguished findings according to whether the study included animal or human subjects. Metrics synthesized included study design, population characteristics, dosage information, and substance use behaviours or outcomes. Our goal was to identify optimal treatment procedure for GLP-1RAs and summarize study findings. Tabular representation of study findings contained more details regarding dosage, timing of GLP-1RA administration, analog-specific characteristics, and individual participant qualities such as baseline substance use.

## 3 Results

We identified eighteen studies, including fourteen animal studies (78%) and four human studies (22%) (Table 2). Six studies (33%) investigated stimulant use, and twelve studies (67%) evaluated opioid use. Research conducted in the United States represented the majority of studies, though four studies (22%) came from Denmark, Sweden, and China. Two studies (11%) operationalized the exposure as the entire GLP1-RA drug class, while the remainder focused on a single analog such as semaglutide, liraglutide, exendin-4, or exenatide, the synthetic version of exendin-4 (Furman, 2007).

**Table 2:** Summary of included studies by relevant descriptive characteristics (n = 18).

**Descriptive characteristics**	**N (%)**
**Studies focused on stimulants**	6 (33%)
Animal studies	5 (28%)
Human studies	1 (5%)
**Studies focused on opioids**	12 (67%)
Animal studies	9 (50%)
Human studies	3 (17%)
**Country of Origin**	
Studies from the US	13 (72%)
Studies from Denmark	2 (11%)
Studies from China	1 (5%)
Studies from multiple countries	1 (5%)
**Exposure of interest**	
All GLP-1RAs	2 (11%)
Exenatide	9 (50%)
Liraglutide	5 (28%)
Semaglutide	2 (11%)

### 3.1 Stimulants

#### 3.1.1 Preclinical studies

Five studies examined GLP-1RA impact on stimulant use in rodents: specifically, impact of semaglutide (one study) or exendin-4 (four studies) treatment on cocaine use, shown in Table 3 (Aranäs et al., 2025, Hernandez et al., 2018, Schmidt et al., 2016, Sørensen et al., 2015, Zhu et al., 2021). All studies found that acute administration of a GLP-1RA, in comparison to a control, reduced cocaine-seeking behaviours and reinstatement of cocaine-seeking behaviours after extinction among stimulant-dependent rodents (Aranäs et al., 2025, Hernandez et al., 2018, Schmidt et al., 2016, Sørensen et al., 2015, Zhu et al., 2021). Zhu et al. (2021) observed these effects after a single injection of exendin-4. Four studies also recorded decreased motivation to administer cocaine among groups treated with a GLP-1RA (Aranäs et al., 2025, Hernandez et al., 2018, Schmidt et al., 2016, Sørensen et al., 2015). These findings were observed across both high and low doses (Aranäs et al., 2025, Hernandez et al., 2018, Schmidt et al., 2016, Sørensen et al., 2015, Zhu et al., 2021). However, Aranäs et al. (2025) investigated differential GLP-1RA dosage effects and found only high doses of semaglutide attenuated acute cocaine use, while no significant effects were found for low dose semaglutide or control groups. Additionally, only Sørensen et al. (2015) investigated chronic stimulant use but found no significant effect of exendin-4 on cocaine seeking behaviours or use.

**Table 3:** Identified preclinical and clinical studies examining stimulant use outcomes (n = 6).

**Author**	**Year**	**Country**	**Objective**	**Sample Size and Subject Characteristics**	**Methods**	**Findings**
***Preclinical studies***						
Aranäs	2025	USA, Sweden	To study the effect of semaglutide on cocaine use, seeking, and related dopamine levels in rats	**N:** 45 **Animal characteristics:** male Sprague-Dawley rats approximately 7 weeks of age and weighing 225–240 g	**Design:** counterbalanced, within subjects design **Year:** N/R **Dosage:** 0.013, 0.026, or 0.039 mg/kg semaglutide administered (s.c.) one hour before experimental session **Substance measure:** active lever response, breakpoints **Statistical methods:** two-way repeated measure ANOVA	Only rats receiving high doses of semaglutide demonstrated a significant reduction in active lever presses during reinstatement, lower motivation to press active levers, and decreased breakpoints
Hernandez	2018	USA	To explore the influence of exendin-4 and GLP-1R activation in the ventral tegmental area on cocaine-seeking in rats	**N:** 52 **Animal characteristics:** male Sprague-Dawley rats weighing 225–250 g	**Design:** counterbalanced, within-subjects design **Year:** N/R **Dosage:** 0.01, 0.1, 0.2 or 3.0 μg/kg exendin-4 administered (i.p.) one hour prior to session **Substance measure:** active lever response **Statistical methods:** two-way ANOVA and Bonferroni post hoc test	Rats treated with exendin-4 exhibited significantly less active lever responses than controls during reinstatement, supported by a significant main effect of treatment
Schmidt & Mietlicki-Baase	2025	USA	To examine the effects of GLP-1R activation on cocaine-seeking in rats and associated neurological activity	**N:** 25 **Animal characteristics:** male Sprague-Dawley rats weighing 225–250 g	**Design:** between-session within-subjects design **Year:** N/R **Dosage:** 0.005 or 0.05 μg exendin-4 immediately before session (intra-ventral tegmental area injection) **Substance measure:** active lever responses, breakpoints, number of infusions **Statistical methods:** one-way repeated measures ANOVA; Tukey's post hoc test	Rats treated with 0.05 μg exendin-4 showed lower active lever presses, breakpoints, and infusions compared to those treated with vehicle (Tukey’s HSD, *p* < 0.05)
Sørensen	2015	Denmark	To investigate the impact of GLP-1R activation on acute and chronic cocaine self-administration as well as cocaine-induced modulation in the brain	**N:** 169 **Animal characteristics:** NMRI and C57BI/6 male mice weighing 20–24 g	**Design:** Latin-square design **Year:** N/R **Dosage:** 10, 30, or 100 μg/kg exendin-4 administered (i.p.) 90–100 min before session; 10 μg/kg exendin-4 administered (i.p.) 60 min before session **Substance measure:** nose pokes **Statistical methods:** one and two-way ANOVA; Bonferroni-corrected comparisons; non-parametric statistics Kruskal-Wallis Analysis of Variance; Mann-Whitney U-tests	Acute treatment of 30 and 100 μg/kg exendin-4 resulted in decreased nose pokes for cocaine (*p* < 0.01 and *p* < 0.05, respectively). Kruskal-Wallis Analysis of Variance indicated no significant effect of exendin-4 long term
Zhu	2021	China	To test the influence of exendin-4 on cocaine-induced conditioned place preference	**N:** 36 **Animal characteristics:** adult male C57BL/6 J mice weighing 18–22 g	**Design:** conditioned place preference **Year:** N/R **Dosage:** 100.0, 30.0, 1.0, and 0.1 μg/kg body weight of exendin-4, administered one hour prior to session (i.p.) **Substance measure:** time spent in cocaine-paired chamber and conditioned place preference score **Statistical methods:** one- and two-way ANOVA; paired *t*-tests; Tukey's, Dunnett's or Bonferroni tests	Both single and repeated administration of exendin-4 (all doses) significantly blocked acquisition, attenuated reinstatement, and facilitated extinction of cocaine-induced conditioned place preference
***Clinical studies***						
Angarita	2021	USA	To investigate if exenatide treatment alters cocaine administration and related subjective experiences among people with cocaine use disorder	**N:** 13 **Population characteristics:** individuals with at least one year diagnosis of cocaine use disorder who were not seeking treatment (1 F:12 M) **Inclusion criteria:** pass a physical exam, electrocardiogram, and lab tests; be 30–55 years old; have self-reported intravenous or smoked cocaine use that exceeds possible use in the present study; have urine benzoylecgonine detection at baseline; have moderate to severe cocaine use disorder as per DSM-5 criteria **Exclusion criteria:** less than one year diagnosis of CUD; history of major psychiatric condition as per DSM-5 (not including cocaine-related diagnoses), medical, or neurological illnesses; fasting glucose level under 70 mg/dl; actively using psychotropic or psychoactive medications; adverse reaction to exenatide; record of medullary thyroid carcinoma or multiple endocrine neoplasia syndrome type 2; pregnant or breastfeeding	**Design:** randomized control trial; double-blind, crossover, within-subject design **Year:** Dec 2014-Jul 2018 **Dosage:** 5 μg exenatide, administered (i.v.) three hours prior to self-administration **Substance measure:** number of infusions, self-reported craving **Statistical methods:** linear mixed modeling analysis, including drug as a within-subjects factor	Neither reported craving nor administration of cocaine differed between exenatide and placebo trials

N/R =  Not recorded

i.p. =  intraperitoneal

s.c. =  subcutaneous

#### 3.1.2 Clinical studies

One study examined the impact of a GLP-1RA on stimulant use in humans (Table 3). Angarita et al. (2024) administered 5 μg of exenatide prior to cocaine self-administration sessions in thirteen people who had chronically used cocaine for at least a year. Participants underwent a training session and two experimental sessions. Results indicated that administration of exenatide was no different from control across both cocaine use measures: the number of infusions and self-reported cravings (Angarita et al., 2024).

### 3.2 Opioids

#### 3.2.1 Preclinical studies

Nine preclinical studies have explored opioid use in opioid-dependent rodents after administration of GLP-1RA analogs liraglutide and exendin-4, as seen in Table 4 (Bornebusch et al., 2019, Douton et al., 2021a, Douton et al., 2021b, Douton et al., 2022, Evans et al., 2022, Urbanik et al., 2022, Urbanik et al., 2024, Zhang et al., 2019, Zhang et al., 2021). One study, by Bornebusch et al. (2019), found no difference between exendin-4 and saline pretreated mice, in contrast to the other eight studies, which found that administration of a GLP-1RA analog attenuated opioid-seeking behaviours or use compared to control (Douton et al., 2021a, Douton et al., 2021b, Douton et al., 2022, Evans et al., 2022, Urbanik et al., 2022, Urbanik et al., 2024, Zhang et al., 2019, Zhang et al., 2021). Three of these eight studies stratified results by high and low opioid-taking rodents and found that liraglutide administration significantly reduced opioid use in both groups, though effects varied according to interactions with liraglutide dose and timing (Evans et al., 2022, Urbanik et al., 2022, Urbanik et al., 2024). Furthermore, Urbanik et al. (2024) and Evans et al. (2022) found greater efficacy in opioid use reduction among high opioid-taking rats in two studies. Five studies also accounted for the effect of time (Douton et al., 2021b, Douton et al., 2022, Evans et al., 2022, Urbanik et al., 2022, Urbanik et al., 2024), and one study by Douton et al. (2021b) found greater differences in opioid use between GLP-1RA and saline-treated rodents during the later hours of the experiment.

**Table 4:** Identified preclinical and clinical studies examining opioid use-related outcomes (n = 12).

**Author**	**Year**	**Country**	**Objective**	**Sample Size and Subject Characteristics**	**Methods**	**Findings**
***Preclinical studies***						
Bornebusch	2019	Denmark	To explore the impact of GLP-1RA exendin-4 on reward, reinforcement, and withdrawal effects from opioid use	**N:** N/R **Animal characteristics:** male B6 mice approximately 7–8 weeks of age; Glp1r^flox/flox^ nestin-Cre^+/-^ KO and WT male mice	**Design:** counterbalanced design for morphine administration sessions; random assignment for remifentanil self-administration sessions **Year:** N/R **Dosage:** 3.2 or 10 μg/kg exendin-4 administered 30 min before experimental session (injection) **Substance measure:** distance traveled and time spent in the morphine-paired chamber, nose pokes, number of vertical jumps **Statistical methods:** one- and three-way ANOVA and Bonferroni correction, paired-sample *t* test	Across all measures, responses from exendin-4 and vehicle treated mice were no different
Douton	2021a	USA	To investigate the effect of exendin-4 on cue- and drug-induced heroin seeking behaviours in rats	**N:** 55 (data from 47) **Animal characteristics:** male Sprague-Dawley rats approximately 90 days of age weighing 300–400 g	**Design:** random assignment; extinction and reinstatement test design **Year:** N/R **Dosage:** 2.4 μg/kg exendin-4 administered (i.p.) once daily for three weeks; one or six hours prior to experimental session **Substance measure:** active spout contacts, mean latency of first contact with active spout **Statistical methods:** Student’s *t*-test, mixed factorial ANOVA, Tukey's post hot test, Kaplan-Meier survival curve, and Log-rank test	1. Chronic treatment of exendin-4 attenuated cue-induced heroin seeking across measures and hours 2. Administration of exendin-4 one hour before session attenuated drug-induced heroin seeking (*p* < 0.0001) but had no impact when administered six hours before
Douton	2021b	USA	To explore how variable liraglutide dosages impact heroin use, heroin seeking behaviours, and adverse side effects	**N:** 16 (experiment 1) **Animal characteristics:** male Sprague-Dawley rats approximately 90 days of age and weighing 300–400 g	**Design:** random assignment; extinction and reinstatement test design **Year:** N/R **Dosage:** 0.1 mg/kg daily liraglutide injection across approximately four weeks (s.c.); one hour before test session on test days **Substance measure:** mean number of infusions, percent change in the number of infusions, rate of heroin use escalation, active spout contacts, mean latency to initiate contact with active spout **Statistical methods:** Student’s *t*-test, mixed factorial and one-way ANOVA, Tukey's post hoc test, Greenhouse-Greisser correction	1. In contrast to rats treated with vehicle, rats treated with liraglutide exhibited lower heroin self-administration and seeking behaviours (*p* < 0.05) 2. Chronic liraglutide treatment significantly inhibited drug-induced heroin seeking but not cue-induced seeking
Douton	2022	USA	To evaluate cue-, drug-, and stress-induced heroin seeking behaviours in rats after liraglutide administration	**N:** 24 (experiment 2) **Animal characteristics:** male Sprague-Dawley rats approximately 90 days of age weighing 300–400 g	**Design:** matched based on number of infusions during acquisition; extinction and reinstatement test design **Year:** N/R **Dosage:** 0.3 mg/kg liraglutide administered six hours before test sessions (s.c.) **Substance measure:** active spout contacts **Statistical methods:** two-way or mixed factorial ANOVA, Tukey's post hoc test, Bonferroni correction	Rats treated with liraglutide had significantly less active spout contacts than vehicle treated rats during drug-induced reinstatement, as well as cue- and stress-induced reinstatement
Evans	2022	USA	To investigate the efficacy of dosage titration of liraglutide on reducing heroin seeking behaviour	**N:** 48 (experiments 1 and 2) **Animal characteristics:** male Sprague-Dawley rats approximately 90 days of age and weighing 300–400 g	**Design:** matched group based on heroin-seeking during acquisition; extinction and reinstatement test design **Year:** N/R **Dosage:** daily injection of 0.06 mg/kg liraglutide increasing to 0.1 mg/kg, 0.3 mg/kg, or 0.6 mg/kg over two weeks; daily injection of 0.3 mg/kg liraglutide (s.c.) **Substance measure:** active spout contacts **Statistical methods:** mixed factorial ANOVA, Newman-Keuls post hoc test	Chronic treatment of liraglutide titrating to a dose of 0.3 mg/kg, but not 0.6 mg/kg, reduced cue-induced heroin seeking in high drug-taking rats and drug-induced heroin seeking in both low- and high-drug taking rats
Urbanik	2024	USA	To test the impact of liraglutide and estrus phase on fentanyl seeking in female rats	**N:** 27 **Animal characteristics:** female Sprague-Dawley rats approximately 90 days old and weighing at least 250 g	**Design:** matched group based on number of infusions during acquisition; extinction and reinstatement test design **Year:** N/R **Dosage:** 0.3 mg/kg liraglutide, administered six hours pretreatment (s.c.) **Substance measure:** infusion attempts (10 active spout contacts) **Statistical methods:** one- and two-way mixed factorial ANOVA, Tukey's post hoc test, unpaired *t*-test, Mauchly's sphericity test, Greenhouse-Geisser correction	1. Acute administration of liraglutide significantly blocked drug-induced fentanyl seeking behaviour for all rats and high-drug takers but not low-drug takers 2. Liraglutide and vehicle treated rats exhibited the same cue-induced fentanyl seeking behaviours
Urbanik	2022	USA	To study the impact of liraglutide on fentanyl seeking behaviours in male rats	**N:** 24 **Animal characteristics:** male Sprague-Dawley rats approximately 90 days of age and weighing at least 300 g	**Design:** matched group based on number of fentanyl infusions during acquisition; extinction and reinstatement test design **Year:** N/R **Dosage:** 0.3 mg/kg liraglutide administered six hours pretreatment (s.c.) **Substance measure:** active spout contacts **Statistical methods:** unpaired *t*-test, two-way mixed factorial ANOVA, Newman-Keuls post hoc test	Acute liraglutide treatment significantly reduced cue- and drug-induced seeking across all subjects, low- and high-drug taking rats
Zhang	2019	USA	To identify the effects of exendin-4 on oxycodone seeking behaviours and use in rats	**N:** 40 **Animal characteristics:** male Sprague-Dawley rats weighing 250–300 g	**Design:** between-session, within-subjects, counterbalanced design; extinction and reinstatement test design **Year:** N/R **Dosage:** 0.3 or 3.0 μg/kg exendin-4, administered 10 min prior to session (i.p.) **Substance measure:** active lever response, number of infusions, breakpoints, total oxycodone infused **Statistical methods:** repeated-measures one and two-way ANOVA, Bonferroni post hoc test	Administration of exendin-4 blocked reinforcing effects and motivation to use oxycodone during self-administration and reinstatement tests
Zhang	2021	USA	To explore the impact of exendin-4 and GLP-1R/Y2R agonist GEP44 on fentanyl seeking behaviors and use in male rats	**N:** 81 **Animal characteristics:** male Sprague-Dawley rats weighing 250–300 g	**Design:** between-session, within-subjects, counterbalanced design; extinction and reinstatement test design **Year:** N/R **Dosage:** 0.072nmol/kg or 0.72nmol/kg exendin-4, 10 min prior to session (i.p.) **Substance measure:** active lever response, number of infusions, total fentanyl infused **Statistical methods:** repeated-measures one- and two-way ANOVA, Bonferroni post hoc test	Exendin-4 groups demonstrated blocked fentanyl self-administration and reinstatement behaviours, supported by significantly reduced total number of infusions during self-administration sessions and reinstatement
***Cohort studies***						
Dai & Radwan	2025	USA	To compare GLP-1RAs to other anti-diabetic medication and their relationship to instances of substance-related hospitalizations among older adults	**N:** 9540 after propensity score matching **Population characteristics:** people newly using anti-T2D drugs with previous diagnosis of T2D and a SUD (46.6% female) **Inclusion criteria:** aged 65 years or older; diagnosis of T2D and an SUD; began GLP-1RA, DPP-4i, or SGLT2i treatment between Jan 2016-Dec 2020; enrollment in Medicaid 1 year prior to first prescription of an anti-diabetic treatment **Exclusion criteria:** previous use of anti-diabetic medication; diagnosis of end stage renal disease; simultaneous prescription of both GLP-1RAs and DPP-4is or SGLT2is	**Design:** retrospective cohort with people newly using, active-comparator design; follow up from first prescription of GLP-1RA until one of the following occurrences: hospitalization due to OUD, death, disenrollment from Parts A, B or D of Medicare, or end of study period **Year:** data collected Jan 2013-Dec 2020 from Medicare data **Dosage:** oral tablet or injection of any GLP-1RA (albiglutide, dulaglutide, exenatide, liraglutide, lixisenatide, semaglutide, tirzepatide) **Substance measure:** hospitalizations for OUD **Statistical methods:** incidence rate, Kaplan-Meier survival analysis, Cox proportional hazards regression, per-protocol principle, Fine-Gray sub-distribution hazard models	1. Compared to people taking DPP-4 inhibitors, people taking GLP-1RAs had a lower risk of opioid-related hospitalization (HR 0.64; 95% CI, 0.43–0.96) 2. No significant difference was found between SGLT2 inhibitors and GLP-1RA groups
Qeadan	2024	USA	To identify the association between GIP or GLP-1RAs and the incidence of opioid overdose/alcohol intoxication among patients with OUD/AUD	**N:** 503,747 **Population characteristics:** adults with a history of OUD or AUD (51.1% female, 48.9% male) **Inclusion criteria:** aged 18 years or older; OUD or AUD diagnosis based on ICD-9-CM, ICD-10-CM, ICD9/10 PSC, SNOMED; **Exclusion criteria:** obtained GIP/GLP-1RA prescription before diagnosis; not meeting full OUD or AUD criteria despite having relevant diagnostic codes	**Design:** retrospective cohort design, with follow up starting 7 days after first prescription of GLP-1RA up to 2 years **Year:** data collected Jan 2014-Sep 2022 from Oracle Cerner Real-World Data **Dosage:** oral tablet or injection of any GLP-1RA (albiglutide, dulaglutide, exenatide, liraglutide, lixisenatide, semaglutide, tirzepatide) **Substance measure:** nonfatal and fatal opioid overdose **Statistical methods:** incidence rate, incidence rate ratio, 95% Wald confidence intervals or 95% exact Poisson confidence intervals, mixed-effects Quasi-Poisson regression model, Cameron and Trivedi’s test	Those with a GIP/GLP-1 RA prescription had a lower likelihood of opioid overdose compared to those without (aIRR [95% CI] = 0.60[0.43, 0.83])
Wang	2024	USA	To explore the association between semaglutide and opioid overdose in people with T2D and OUD	**N:** 5580 after propensity matching **Population characteristics:** people with diagnoses of both T2D and OUD **Inclusion criteria:** T2D and OUD diagnosis; prescription for anti-diabetic medication between Dec 2017-Jun 2023; history of obesity, hypertension, hypercholesterolemia, hyperlipidemia, heart diseases, or stroke **Exclusion criteria:** underwent bariatric surgery; diagnosed with pancreatitis, type 1 diabetes, thyroid cancer, or gastroparesis	**Design:** retrospective cohort, with follow up from first prescription of semaglutide until one of the following occurrences: opioid overdose, death, loss to follow-up, or 12 months **Year:** data collected Dec 2017-Jun 2023 from TriNetX Analytics Platform **Dosage:** oral tablet or injection of semaglutide **Substance measure:** nonfatal and fatal opioid overdose **Statistical methods:** Cox proportional hazard and Kaplan-Meier survival analysis	Those with a semaglutide prescription, compared to other anti-diabetic drugs (insulin, metformin, DPP-4 inhibitors, SGLT2 inhibitors, sulfonylureas, thiazolidinediones, or other GLP-1RAs), had a significantly lower risk of opioid overdose (HRs ranging from 0.32 (95% CI, 0.12–0.89) to 0.58 (95% CI, 0.38–0.87))

N/R =  Not recorded

i.p. =  intraperitoneal

s.c. =  subcutaneous

DPP-4i =  dipeptidyl peptidase-4 inhibitor

SGLT2i =  sodium-glucose transporter 2 inhibitor

#### 3.2.2 Cohort studies

Three retrospective cohort studies explored the impact of GLP-1RAs on opioid use outcomes in humans with a diagnosis of opioid use disorder, shown in Table 4 (Dai et al., 2025, Qeadan et al., 2025, Wang et al., 2024). Specifically, these studies compared opioid-related overdoses and hospitalizations across two years in large cohorts with and without a GLP-1RA prescription. Both studies investigating overdose as the substance use-related outcome found GLP-1RA to be protective: the study by Qeadan et al. (2025) (n = 503,747) found any GLP-1RA analog to be protective, and the study by Wang et al. (2024) (n = 5580) found semaglutide to be protective. These results were robust to stratifying by chronic conditions, obesity, and type II diabetes (Qeadan et al., 2025, Wang et al., 2024). However, the study by Dai & Radwan et al. (2025) (n = 9540), conducted among people with type 2 diabetes, reported that those taking GLP-1RAs only had a lower risk of opioid-related hospitalizations when compared to people taking dipeptidyl peptidase-4 inhibitors, but not to those taking sodium-glucose transport 2 inhibitors, two other classes of medications prescribed to treat type 2 diabetes.

## 4 Discussion

Both preclinical and cohort studies, but not the clinical study for cocaine, support GLP-1RA administration as a treatment for stimulant and opioid dependence, particularly GLP-1RAs exenatide or exendin-4, liraglutide, and semaglutide (Aranäs et al., 2025, Dai et al., 2025, Douton et al., 2021a, Douton et al., 2021b, Douton et al., 2022, Evans et al., 2022, Hernandez et al., 2018, Qeadan et al., 2025, Schmidt et al., 2016, Sørensen et al., 2015, Urbanik et al., 2022, Urbanik et al., 2024, Wang et al., 2024, Zhang et al., 2019, Zhang et al., 2021, Zhu et al., 2021). Compared to both placebo and active control groups, GLP-1RAs were deemed an effective treatment across most trials establishing baseline substance use, motivation to use substances, and, in animals, reinstatement of drug use after extinction (Aranäs et al., 2025, Douton et al., 2021a, Douton et al., 2021b, Douton et al., 2022, Evans et al., 2022, Hernandez et al., 2018, Schmidt et al., 2016, Sørensen et al., 2015, Urbanik et al., 2022, Urbanik et al., 2024, Zhang et al., 2019, Zhang et al., 2021, Zhu et al., 2021). Some studies observed greater impact to drug use in high drug-taking animals compared to low takers, suggesting prior drug use patterns may influence the success of GLP-1RA treatment (Evans et al., 2022, Urbanik et al., 2024). Additionally, variation in efficacy depending on the dosage of GLP-1RA aligns with other research findings, which suggest that there is an optimal dosage of GLP-1RA treatments that balances more significant reductions in drug use and adverse effects (Bettge et al., 2016, Ghidewon et al., 2022, Kanoski et al., 2012). Thus, the lack of significant results from Bornebusch et al. (2019) might be explained by the low dose of exendin-4 and short incubation period between injection and self-assessment. Overall, consistent results associating GLP-1RA treatment with reduced stimulant and opioid use suggest that this effect is reproducible across contexts and populations (Aranäs et al., 2025, Douton et al., 2021a, Douton et al., 2021b, Douton et al., 2022, Evans et al., 2022, Hernandez et al., 2018, Schmidt et al., 2016, Sørensen et al., 2015, Urbanik et al., 2022, Urbanik et al., 2024, Zhang et al., 2019, Zhang et al., 2021, Zhu et al., 2021). These findings mirror the literature on GLP-1RA treatment for alcohol and other substance use disorders, which also support a reduction in alcohol and substance intake among both rodents and humans (Arillotta et al., 2024, Falk et al., 2023, Hendershot et al., 2025, Lengsfeld et al., 2023, Quddos et al., 2023, Thomsen et al., 2017, Tuesta et al., 2017, Wium-Andersen et al., 2022, Yammine et al., 2021). GLP-1RA interaction with reward pathways and contribution to muted cravings may explain this generalized effect across alcohol and substance use disorders (Arillotta et al., 2024, Brunchmann et al., 2019, Chuong et al., 2023).

Despite these preliminary findings, more research is needed to corroborate the results among human participants. Across studies investigating both opioid and stimulant use, only a few clinical studies exist, none of which establish causality (Angarita et al., 2024, Dai et al., 2025, Qeadan et al., 2025, Wang et al., 2024). The single study we identified that explored stimulant use among humans used a small sample size and only followed participants over a short period of two days (Angarita et al., 2024). Only three studies followed substance use patterns with GLP-1RA administration for longer periods up to two years, highlighting that potential differences arising from chronic substance use remain relatively unexplored (Dai et al., 2025, Qeadan et al., 2025, Wang et al., 2024). Ensuring that GLP-1RAs are safe and effective as a substance use disorder treatment long-term is essential since substance use disorder and related outcomes, such as incidences of overdose, can be chronic (Response to the Opioid Overdose Crisis in Vancouver Coastal Health, 2018; “The ASAM National Practice Guideline for the Treatment of Opioid Use Disorder,” 2020). Furthermore, studies investigating opioid use relied on electronic medical records, meaning results only captured data on the most severe outcomes, such as overdose (Dai et al., 2025, Qeadan et al., 2025, Wang et al., 2024). This methodology also required substance use metrics to reflect formal diagnosis of a substance use disorder and did not include less severe substance use. Published literature has shown that people consuming higher amounts of alcohol experience greater reduction in alcohol use after GLP-1RA administration compared to low alcohol takers (Ghidewon et al., 2022). For opioid use studies, this means more investigation is needed to establish if the same trends from GLP-1RA treatment appear for all opioid use, specifically giving attention to less severe substance use outcomes and trends for people who use opioids but are not diagnosed with opioid use disorder. Improved understanding of underlying biological mechanisms may reveal why treatment effectiveness might vary.

Within this review, two studies evaluated all GLP-1RA analogs as a treatment for substance use disorder (Dai et al., 2025, Qeadan et al., 2025), while all other studies focused solely on exenatide, liraglutide, or semaglutide as an exposure (Angarita et al., 2024, Aranäs et al., 2025, Bornebusch et al., 2019, Douton et al., 2021a, Douton et al., 2021b, Douton et al., 2022, Evans et al., 2022, Hernandez et al., 2018, Schmidt et al., 2016, Sørensen et al., 2015, Urbanik et al., 2022, Urbanik et al., 2024, Wang et al., 2024, Zhang et al., 2019, Zhang et al., 2021, Zhu et al., 2021). Among these three GLP-1RAs, only two studies captured the effects of semaglutide in isolation (Sørensen et al., 2015, Wang et al., 2024). Despite its low representation in this review, semaglutide is the most commonly prescribed GLP-1RA for weight loss and type 2 diabetes, potentially due to its effectiveness and ease of use (Ukhanova et al., 2025). This analog has been shown to bond well with the GLP-1 receptors and has a long half-life of 160 h, which allows for glycemic control, reduced risk of cardiovascular events, and reduced burden on the patient to readminister medication (Schuckit, 2009). Distinct from other GLP-1RA analogs, semaglutide is one of the few long-acting agents in this class and can be administered orally, as opposed to by subcutaneous injection, improving ease of use and treatment adherence (Schuckit, 2009). Semaglutide’s greater success in treatment for its approved uses suggests that there could be differences in efficacy for treating substance use disorders as well. Findings from Wang et al. (2024) support that semaglutide has protective factors against opioid overdose compared to other GLP-1RAs, as demonstrated by a significant hazard ratio for fatal and nonfatal overdose less than one. However, more evidence is needed elucidating how differing pharmacokinetics between GLP-1RA analogs can lead to variable substance use outcomes.

Though this review used a standardized methodology to ensure applicability and relevance of included literature, some limitations remain. For instance, only one reviewer screened search results and extracted data; this study does not achieve the rigor of a systematic review. It is possible that studies were inadvertently excluded; however, given the small number of relevant studies identified, human error pertaining to data extraction and study identification is minimized. Next, literature could only be included if identified through search results from journal databases. Potentially non-exhaustive search terms and lack of a grey literature search might have excluded relevant literature. Relying on English studies alone could have further contributed to restricted search results and limited findings overall.

## 5 Conclusions

In all, the studies examined in the present review suggest that administration of a GLP-1RA attenuates stimulant and opioid use across animal and human subjects, particularly among those with higher levels of substance use. However, more research is needed with attention towards clinical studies, specifically randomized clinical trials and long-term follow-up, in order to establish efficacy and safety across changing contexts and populations. Future studies should also investigate individual differences between GLP-1RA analogs to determine which are best suited for stimulant and opioid use disorder treatment.

## CRediT authorship contribution statement

**Marisa Leach:** Writing – review & editing, Writing – original draft, Methodology, Investigation, Conceptualization. **Megan E. Marziali:** Writing – review & editing, Supervision, Methodology, Conceptualization. **Surita Parashar:** Writing – review & editing, Conceptualization. **Greg Bondy:** Writing – review & editing, Conceptualization. **Silvia Guillemi:** Writing – review & editing, Conceptualization. **Marianne Harris:** Writing – review & editing, Conceptualization. **Mark Hull:** Writing – review & editing, Conceptualization. **Sarah Stone:** Writing – review & editing, Conceptualization. **Katherine W. Kooij:** Writing – review & editing, Conceptualization. **Robert S. Hogg:** Writing – review & editing, Supervision, Conceptualization.​​​​​ **Julio S.G. Montaner:** Writing – review & editing, Supervision, Conceptualization.

## Funding

This research did not receive any specific grant from funding agencies in the public, commercial, or not-for-profit sectors.

## Declaration of Competing Interest

The authors declare that they have no known competing financial interests or personal relationships that could have appeared to influence the work reported in this paper.
