# Supplementary material for: A rapid review of the effects of GLP-1 receptor agonists on opioid and stimulant use-related outcomes
Source: Drug Alcohol Depend Rep. 2026 Apr 24;19:100440. doi: 10.1016/j.dadr.2026.100440 (PMC13156756; doi:10.1016/j.dadr.2026.100440)
Supplement: Table S1 — Supplementary material [file mmc1.docx]

**Supplemental Tables.**

**Table S1.** Example search conducted in Scopus.

| **Database** | **Complete search strategy** |
| --- | --- |
| Scopus | TITLE-ABS-KEY((agonists, glp 1r) OR (agonists, glp1r) OR (semaglutide) OR (lixisenatide) OR (albiglutide) OR (dulaglutide) OR (liraglutide) OR (exenatide) OR (ozempic) OR (wegovy) OR (trulicity) OR (victoza) OR (saxenda) OR (mounjaro) OR (adlyxin) OR (eperzan) OR (byetta) OR (bydureon) OR (zepbound) OR (tanzeum) OR (rybelsus) OR (soliqua) OR (xultrophy)) AND TITLE-ABS-KEY((opioid*) OR (opiate*) OR (cocaine) OR (fentanyl) OR (oxycodone) OR (stimulant*) OR (heroin) OR (morphine) OR (methamphetamine*) OR (amphetamine*)) |

**Table S2.** Data extraction table template.

| **Extraction variable** | **Study 1** | **Study 2 (…)** |
| --- | --- | --- |
| Year |  |  |
| Author |  |  |
| Title |  |  |
| Country |  |  |
| Aim |  |  |
| Dataset |  |  |
| Years of data collection |  |  |
| Total participants/subjects |  |  |
| Study population |  |  |
| Inclusion criteria |  |  |
| Exclusion criteria |  |  |
| Gender identification |  |  |
| Animal Model |  |  |
| Species |  |  |
| Sex |  |  |
| Strain |  |  |
| Age |  |  |
| Weight |  |  |
| Methods |  |  |
| Study design |  |  |
| Exposure |  |  |
| Number of participants/subjects |  |  |
| Drug/chemical name |  |  |
| Dose concentration, duration, frequency |  |  |
| Route of administration (i.p., s.c., injection, inhalation) |  |  |
| Vehicle |  |  |
| Outcome |  |  |
| Measure of opioid/stimulant use (number of administrations, volume of substance) |  |  |
| Measure of opioid/stimulant use seeking behaviors (pedal pressing, nose touches) |  |  |
| Opioid/stimulant craving |  |  |
| Overdose |  |  |
| Hospitalization |  |  |
| Statistical methods |  |  |
| Findings |  |  |
